# Surface Plasmon Resonance Sensor Based on Core-Shell Fe_3_O_4_@SiO_2_@Au Nanoparticles Amplification Effect for Detection of T-2 Toxin

**DOI:** 10.3390/s23063078

**Published:** 2023-03-13

**Authors:** Lirui Fan, Bin Du, Fubin Pei, Wei Hu, Aijiao Guo, Zihao Xie, Bing Liu, Zhaoyang Tong, Xihui Mu, Wenyuan Tan

**Affiliations:** 1 School of Chemical Engineering, Sichuan University of Science and Engineering, Zigong 643000, China; 2 State Key Laboratory of NBC Protection for Civilian, Beijing 102205, China

**Keywords:** surface plasmon resonance, Fe_3_O_4_@SiO_2_@AuNPs nanocomposite, T-2 toxin detection, SPR-based sensor

## Abstract

In this paper, a core-shell based on the Fe_3_O_4_@SiO_2_@Au nanoparticle amplification technique for a surface plasmon resonance (SPR) sensor is proposed. Fe_3_O_4_@SiO_2_@AuNPs were used not only to amplify SPR signals, but also to rapidly separate and enrich T-2 toxin via an external magnetic field. We detected T-2 toxin using the direct competition method in order to evaluate the amplification effect of Fe_3_O_4_@SiO_2_@AuNPs. A T-2 toxin–protein conjugate (T2-OVA) immobilized on the surface of 3-mercaptopropionic acid-modified sensing film competed with T-2 toxin to combine with the T-2 toxin antibody–Fe_3_O_4_@SiO_2_@AuNPs conjugates (mAb-Fe_3_O_4_@SiO_2_@AuNPs) as signal amplification elements. With the decrease in T-2 toxin concentration, the SPR signal gradually increased. In other words, the SPR response was inversely proportional to T-2 toxin. The results showed that there was a good linear relationship in the range of 1 ng/mL~100 ng/mL, and the limit of detection was 0.57 ng/mL. This work also provides a new possibility to improve the sensitivity of SPR biosensors in the detection of small molecules and in disease diagnosis.

## 1. Introduction

Surface plasmon resonance (SPR) sensing technology is a high-tech method based on the noble metal surface plasmon resonance phenomenon [1,2]. Because of its advantages [3] of real-time online monitoring, label-free sensing and high sensitivity, the SPR senor has been applied to various fields such as environmental monitoring [4], new drug research and development [5], and food science and biology [6]. Essentially, the SPR sensor is extremely sensitive to changes in the refractive index of a medium on the surface of noble metal film [7]. The refractive index of a noble metal film surface varies with the mass of attached biomolecules, so dynamic change in the SPR resonance curve can reflect the interaction between biomolecules [8,9]. When analytes are macromolecules, such as IgG [10], growth factor-binding protein 7 (IGFBP7) [11] and tumor necrosis factor alpha (TNF-α) [12], direct detection methods based on SPR sensors provide good sensitivity. However, when the analytes are smaller-molecular-weight molecules, including tetracycline (TC) [13], estradiol (E2) [14] and chlorpyrifos (CPF) [15], the analytes are injected into the sample chamber on the surface of the ligand or antibody coupled-monolayer sensing membrane and interact with specific molecules immobilized on the surface of its sensing chip. Owing to its molecular weight being too small to obviously change the refractive index on a nearby metal chip surface [16], the SPR signal response is weak and has insufficient sensitivity to detect small molecules.

It was reported in several papers that nanoparticles [17] were used to improve the sensitivity of SPR sensors including; these nanoparticles included AuNPs [18], AgNPs [19], PdNPs [20], PtNPs [21] and magnetic nanoparticles (MNPs) [22]. These metal nanoparticles have unique optical properties and good biocompatibility, and can also be coupled with the propagating plasma on the surface of the SPR sensing chip, significantly enhancing the SPR response. Considering their large molecular mass and their high refractive index, magnetic nanoparticles can effectively amplify SPR signals. Gold magnetic nanoparticles have attracted much attention in the field of sensors. They have great application prospects in enhancing the sensitivity of SPR sensors due to their dual functions [23,24,25] of magnetic and optical properties. The main reasons for this enhancement are the larger molecular weight and higher refractive index of gold magnetic nanoparticles. Additionally, the gold magnetic nanoparticles can quickly separate and enrich the crucial component in complex samples via an external magnetic field. Today, there are few studies [26] on the construction of SPR sensors based on core-shell Fe_3_O_4_@Au nanoparticles to detect small molecular substances.

**Figure 1:**
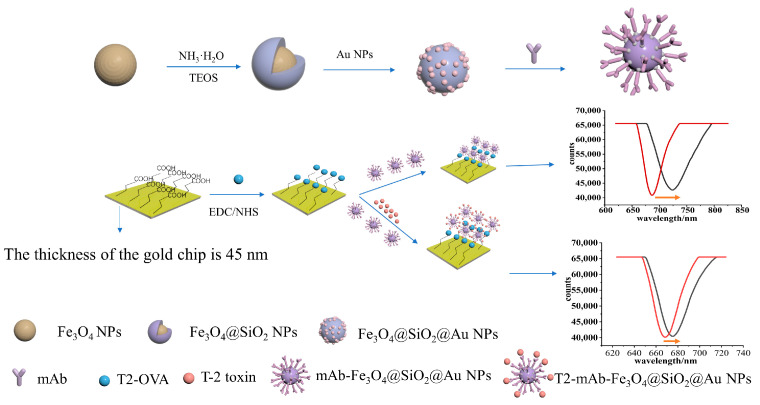
A flow chart of synthesis process of Fe_3_O_4_@SiO_2_@AuNPs and a schematic diagram of T-2 toxin detection principle.

T-2 toxin [27,28,29] is the most toxic type-A trichothecene mycotoxin produced by Fusarium. T-2 toxin even has an unavoidable deleterious influence [30] to health in humans and animals though different routes of exposure, such as skin, air and in the food chain. Acute T-2 toxin poisoning leads to severe skin and eye irritation, cough, coagulopathy, nausea and vomiting, diarrhea, chest pain, abdominal pain and even death. Therefore, in order to address the potential threat of T-2 toxin, it is necessary to develop a highly sensitive method for the detection of T-2 toxin. Herein, a SPR signal amplification strategy based on core-shell Fe_3_O_4_@SiO_2_@Au nanoparticles was developed for the quantification of T-2 toxin using a direct competition method. As shown in Figure 1, T2-OVA was fixed on the surface of a 3-mercaptopropionic acid-modified sensing film. A monoclonal antibody against T-2 toxin-Fe_3_O_4_@SiO_2_@Au nanoparticles (mAb-Fe_3_O_4_@SiO_2_@AuNPs) was not only regarded as an amplification reagent to amplify the SPR signal, but it could also capture T-2 toxin in actual complex samples via an external magnetic field. T2-OVA was bound to mAb-Fe_3_O_4_@SiO_2_@AuNPs and competed competed with T-2 toxin. With the decrease in T-2 toxin concentration, more conjugates of mAb-Fe_3_O_4_@SiO_2_@AuNPs combined with T2-OVA on the sensing film surface, which lead to an increase in the change value of the resonance wavelength. Thus, an SPR sensor based on Fe_3_O_4_@SiO_2_@Au nanoparticles as signal amplifying elements was developed, which provided a practical and theoretical basis for the detection of small molecules in the future.

## 2. Experimental Procedure

Details of the reagents, instruments and construction of the SPR sensor can be found in the Supplementary Materials.

**Figure 2:**
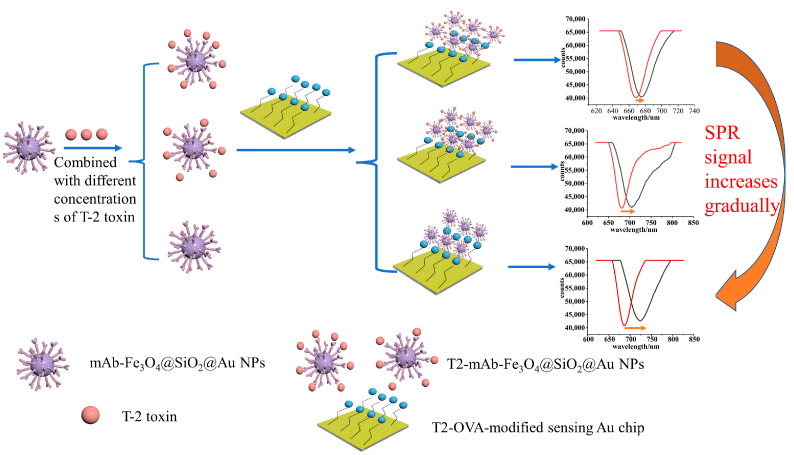
A schematic diagram of the experimental procedure of detecting T-2 toxin based on Fe_3_O_4_@SiO_2_@Au nanoparticle amplification SPR sensor.

In this study, a direct competition method was used to detect T-2 toxin, as shown in Figure 2. Firstly, the synthesized Fe_3_O_4_@SiO_2_@AuNPs nanocomposites were modified with T-2 toxin monoclonal antibody via electrostatic adsorption and stored in a 4 °C cabinet for later use. Then, the gold chip was modified with a carboxyl group through a gold sulfhydryl bond, and then, T2-OVA was modified on the surface of the gold chip through an amide bond. The gold chip modified with T2-OVA was immersed in PBS buffer solution at 4 °C for later use. Then, the mAb-Fe_3_O_4_@SiO_2_@AuNPs nanocomposites were combined with different concentrations of T-2 toxin and injected into the surface of the gold chip modified with T2-OVA for sensing detection. Before the detection of the sample, the PBS buffer solution was injected into the sample chamber to record the signal value of the resonance wavelength (λ1) when the resonance wavelength was no longer moving, and then, the Fe_3_O_4_@SiO_2_@Au nanoparticles, combined with a certain concentration of T-2 toxin, were introduced for sensing detection. When the detection time was 15 min, the signal value of the resonance wavelength (λ2) was recorded, and the change value of the resonance wavelength Δλ (Δλ = λ2 − λ1) was reflected the SPR signal caused by the concentration of T-2 toxin. The lower the concentration of T-2 toxin in the solution, the higher the SPR signal value. 

### 2.1. Synthesis of Fe_3_O_4_@SiO_2_@Au

The Fe_3_O_4_ nanoparticles were prepared using hydrothermal methods [31]. In brief, PSSMA, FeCl_3_·6H_2_O and sodium acetate, one-by-one, were dissolved in ethylene glycol. Then, the mixtures were poured into a Teflon-lined stainless autoclave and heated at 200 °C for 10 h. After cooling to room temperature, a black solution was obtained and washed three times with ethanol and pure water, respectively. The Fe_3_O_4_ nanoparticles were dried at 60 °C for 8 h and stored for further use.

A total of 240 mg Fe_3_O_4_NPs was ultrasonically dispersed in 24 mL ultrapure water. A total of 160 mL ethanol and 8 mL ammonia solution were added into the mixture and stirred mechanically. At the beginning of stirring, 160 μL, 140 μL and 120 μL TEOS were added every 20 min, and then, the reaction continued for an hour. The resulting Fe_3_O_4_@SiO_2_NPs [32] were immediately centrifuged and redispersed in water to form a 10 mg/mL solution. Functionalization of Fe_3_O_4_@SiO_2_NPs with APTES was performed prior to the deposition of gold nanoparticles. A total of 10 mL Fe_3_O_4_@SiO_2_NPs was added to 50 mL ethanol, and 60 mL APTES was dripped into the solution while mechanically stirring for 10 h. Additionally, 100 mg NH_2_-modified Fe_3_O_4_@SiO_2_NPs was added into 10 mL water, and 200 mL Au NPs was added while stirring for 10 h. Fe_3_O_4_@SiO_2_@Au [33] was obtained and washed three times with pure water, and then, redispersed in ultrapure water for further use. And AuNPs were prepared by citric acid reduction method [34]. In brief, 1 mL 1% HAuCl4 was dissolved in 99 mL ultrapure water via oil bath heating to 100 °C, and 1% sodium citrate was added to the solution for 15 min. The Au NPs were obtained and stored at 4 °C.

### 2.2. Functionalization of the Sensing Chip

First, piranha solution (H_2_SO_4_:H_2_O_2_ = 7:3, *v*/*v*) was dropped onto the surface of gold film for ten minutes to eliminate impurity. The gold film was washed with abundant water, ultrasonically activated with ethanol for ten minutes and dried with nitrogen. Then, the activated chip was added to 1 mol/L 3-mercatopropionic acid solution for 12 h. Additionally, the 3-mercatopropionic acid-modified chip was rinsed with lots of ultrapure water, dried with nitrogen, and then, activated using EDC/NHS (10 mg/mL, mixed in equal volume) for 40 min. Additionally, the activated chip was cleaned with lots of ultrapure water, dried with nitrogen, and functionalized using 200 μL T2-OVA (200 μg/mL) for two hours. After washing off non-specific adsorption with a large amount of PBS and water, 200 μL ethanolamine (1 mol/L, pH = 8.5) was added to seal the unbound site for 1 h. Finally, the functionalized chip was rinsed with ultrapure water and dried with nitrogen for further use. 

### 2.3. Preparation of mAb-Fe_3_O_4_@SiO_2_@AuNPs

Monoclonal antibodies against T-2 toxin and Fe_3_O_4_@SiO_2_@AuNPs were diluted with PBS (10 mM, pH = 7.4). A total of 100 μL T2-mAb was mixed with 100 μL Fe_3_O_4_@SiO_2_@AuNPs in 4 °C for 10 h. Then, the mixture was washed three times with PBST, blocked with 1% BSA for one hour and stored at 4 °C for further use.

### 2.4. Immunoassay

T-2 toxin was dissolved in ethanol and diluted in PBS. Different concentrations of T2 toxin (500, 100, 70, 50, 25, 10, 1 and 0.1 ng/mL) and identical concentrations of mAb-Fe_3_O_4_@SiO_2_@Au NPs were mixed for one hour at room temperature. Then, the conjugates (T2-mAb-Fe_3_O_4_@SiO_2_@AuNPs) were washed three times with PBST and finally dispersed in PBS. Meanwhile, CH_2_I_2_ was added dropwise to the optical prism to couple the functional chip, and the sample chamber was fixed on the chip. Additionally, the conjugates were injected into a sample chamber for the reaction. Notably, every sample was reacted for fifteen minutes, and PBS was added to obtain a baseline before every sample was added.

### 2.5. Specificity and Repeatability

To estimate the specificity of the SPR biosensor, a series of structural and functional analogues of T-2 toxin, such as deoxynivalenol, fumonisin B1, aflatoxin B1 and BSA were used to for detection. These analogues were dissolved in ethanol and diluted in 10 mmol/L PBS to form a concentration of 10 ng/mL. Additionally, the detection methods for these analogues were consistent with those for T-2 toxin. Meanwhile, in order to investigate the repeatability of the sensor, different concentrations of T-2 toxin (70 ng/mL, 50 ng/mL and 10 ng/mL) were selected to carry out detection three times.

### 2.6. Analysis of Actual Samples

Milk was purchased from the local supermarket and was used to prepare actual samples. The milk was diluted 10 times with PBS, and different concentrations of T-2 toxin (25 ng/mL, 10 ng/mL and 3 ng/mL) were prepared by adding standard T-2 toxin. Rainwater and soil were taken from the local area, diluted with PBS and centrifuged at 10,000 r/min for 5 min. After repeating these steps three times, the T-2 toxin standard was added to the supernatant to prepare samples with different concentrations (25 ng/mL, 10 ng/mL and 3 ng/mL) for detection. 

## 3. Results and Discussion 

### 3.1. Characterization of Fe_3_O_4_@SiO_2_@AuMNPs

**Figure 3:**
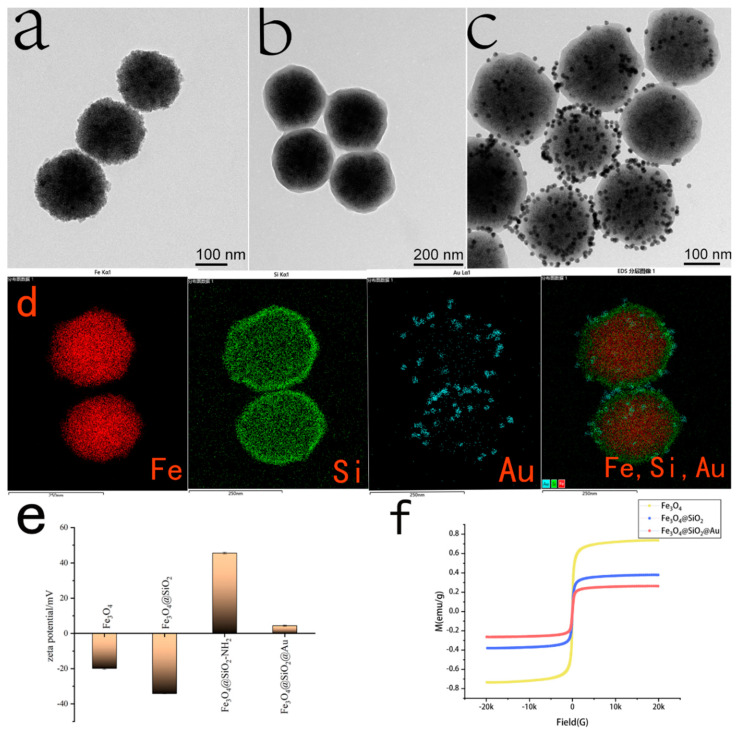
Characterization of nanoparticles: TEM images of (**a**) Fe_3_O_4_NPs, (**b**) Fe_3_O_4_@SiO_2_NPs and (**c**) Fe3O4@SiO2@AuNPs. (**d**) The elemental mapping images of Fe_3_O_4_@SiO_2_@Au MNPs based on Fe, Si and Au elements. (**e**) The zeta potential of Fe_3_O_4_NPs, Fe_3_O_4_@SiO_2_NPs, Fe_3_O_4_@SiO_2_−NH_2_ and Fe_3_O_4_@SiO_2_@AuNPs. (**f**) The hysteresis loop of Fe_3_O_4_NPs, Fe_3_O_4_@SiO_2_NPs and Fe_3_O_4_@SiO_2_@AuNPs.

The morphology and size of nanoparticles, including Fe_3_O_4_NPs, Fe_3_O_4_@SiO_2_NPs and Fe_3_O_4_@SiO_2_@AuNPs, obtained in each step were confirmed via TEM (Figure 3a–c). As shown in Figure 3a, Fe_3_O_4_NPs have a diameter of about 150 nm and good dispersibility. In Figure 3b, a thin layer of silica was successfully coated on the surface of Fe_3_O_4_ NPs, forming Fe_3_O_4_@SiO_2_ NPs with a core-shell structure and a particle size of about 180 nm. Because of the good biocompatibility and large specific surface area of silica, it was used to connect the magnetic core (Fe_3_O_4_NPs) and provide a surface for AuNP sedimentation. After functionalization of the Fe_3_O_4_@SiO_2_ NPs with APTES, the strong affinity between the positive charge of the amino group in APTES and the negative charge of AuNPs allowed AuNPs to deposit on the surface of Fe_3_O_4_@SiO_2_ NPs to form Fe_3_O_4_@SiO_2_@Au MNPs with a core-shell structure. Additionally, in Figure 3c, it is shown that a large number of AuNPs were successfully deposited on the surface of Fe_3_O_4_@SiO_2_NPs; the particles size of Fe_3_O_4_@SiO_2_@Au MNPs was about 220 nm, and they had good dispersibility. To prove the uniformity of the particle size distribution, we provided multiple TEM pictures in Figure S5.

To further confirm the distribution and atomic composition of Fe_3_O_4_@SiO_2_@Au MNPs, an EDX-TEM image was used to analyze the nanoparticles. In Figure 3d, different elements are distinguished by different colors, where red, green and blue represent iron, silicon and gold atoms, respectively. The successful deposition of gold nanoparticles onto the surface of Fe_3_O_4_@SiO_2_@Au MNPs can be clearly observed.

Zeta potential is an important index for characterizing the stability of a dispersion system. The zeta potentials of Fe_3_O_4_NPs, Fe_3_O_4_@SiO_2_NPs, Fe_3_O_4_@SiO_2_-NH_2_ and Fe_3_O_4_@SiO_2_@AuNPs were detected, and the results are shown in Figure 3e. It is generally believed that the Zeta potential of nanoparticles, at about 20 mV, can be used as direct proof of their good dispersion. This is because Fe_3_O_4_NPs are synthesized by the reduction of sodium citrate, therefore it was modified with carboxyl groups. Additionally, the zeta potential value detected was negative, and the value was −19.9 mV, indicating that Fe_3_O_4_NPs are well dispersed. Fe_3_O_4_@SiO_2_ NPs have a higher negative potential after being coated with silica, and their value was −34.2 mV; this was caused by the negatively charged hydroxyl groups on the surface of silica, indicating that the dispersion of Fe_3_O_4_@SiO_2_ NPs was better than that of Fe_3_O_4_NPs. After reacting with APTES, the zeta potential of Fe_3_O_4_@SiO_2_-NH_2_ increased to a positive potential (+45.5 mV). Because the positively charged amino group replaced a part of the negatively charged hydroxyl group, the nanoparticles showed a positive potential and good dispersion. The Fe_3_O_4_@SiO_2_@Au nanoparticles maintained a steady state mainly through the charge effect and van der Waals forces, in which the charge effect occupied the main position. Due to their large particle size, the charge effect between the particles was strong, which ultimately made the dispersion of particles unstable, and the potential value was 4.4 mV.

Magnetic properties played a vital role in the simple recovery of samples in terms of reuse. The Fe_3_O_4_ core provided magnetic properties to Fe_3_O_4_@SiO_2_@Au MNP composites, and the hysteresis loop of the material was tested using VSM, as shown in Figure 3f, the hysteresis loops of Fe_3_O_4_NPs, Fe_3_O_4_@SiO_2_NPs and Fe_3_O_4_@SiO_2_@AuNPs were all S-type and strictly symmetrical about the origin, the magnetization and demagnetization curves were completely coincident, the coercivity was zero, and there was no hysteresis. When the magnetic strength was zero, the remanence was zero, indicating that the prepared composite nanoparticles were superparamagnetic. The saturation magnetization of Fe_3_O_4_NPs, Fe_3_O_4_@SiO_2_NPs and Fe_3_O_4_@SiO_2_@AuNPs gradually decreased, and the values were 73.5 emu/g, 37.9 emu/g and 26.3 emu/g, respectively. It can be seen that as the surface was coated with SiO_2_ and AuNPs, the magnetization intensity of the core-shell nanoparticles decreased. In order to verify the magnetic recovery ability of the composite material in the aqueous solution, a certain amount of Fe_3_O_4_@SiO_2_@AuNPs was ultrasonically dispersed into the aqueous solution. The Fe_3_O_4_@SiO_2_@AuNPs were quickly separated from the water within 60 s under the action of an external magnetic field. Although the magnetic properties of the composite Fe_3_O_4_@SiO_2_@AuNPs were greatly reduced, they were sufficient for magnetic recovery, so the composite has possibilities for practical application.

### 3.2. Functionalization of the Sensing Chip

**Figure 4:**
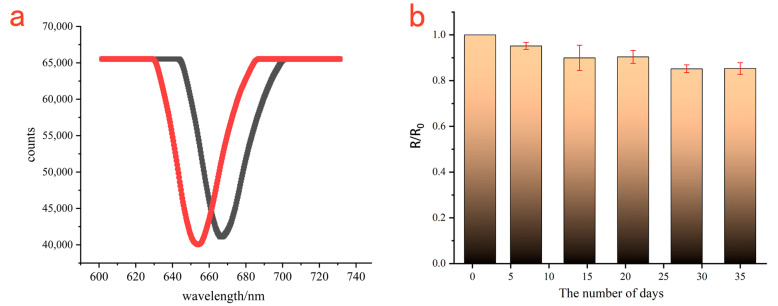
The resonance spectra of PBS on the SPR sensing chip before and after modification with T2-OVA (**a**); the stored stability of T2-OVA-modified Au film (**b**).

3-mercatopropionic acid was used to immobilize T2-OVA, considering that the large number of carboxyl groups that exist on its surface are favorable for immobilizing T2-OVA. The optimal concentration of T2-OVA was selected by observing shifts in resonance wavelength when different concentrations of T2-OVA were injected into the chamber on the surface of functionalized chip film. It is obvious from Figure S1 that the T2-OVA immobilized at a concentration of 200 μg/mL nearly attained saturation. As shown in Figure 4, the resonance wavelength was 657.74 nm based on the chip film without T2-OVA, and further, the resonance wavelength was 677.32 nm when T2-OVA was injected at a concentration of 200 μg/mL. So, the maximum resonance wavelength redshifted by 13.58 nm after T2-OVA was fixed on the chip. Meanwhile, the T2-OVA modified Au film was evaluated for its stored stability. The T2-OVA-modified Au film was immersed in PBS buffer and stored at 4 °C. A total of 15 μg/mL monoclonal antibody was detected every 7 days based on the T2-OVA-modified Au film. The average value of the change in the resonance wavelength detected on the first day was referred to as R_0_, the average value of the change in the resonance wavelength detected over the following days was referred to as R, and R/R_0_ was used to represent the stored stability of the sensing chip. The relationship between R/R_0_ and days is shown in Figure 4b. It is observed that the biological activity of the chip was still more than 80% on the 35th day, indicating that the T2-OVA modified chip had a good stability.

**Figure 5:**
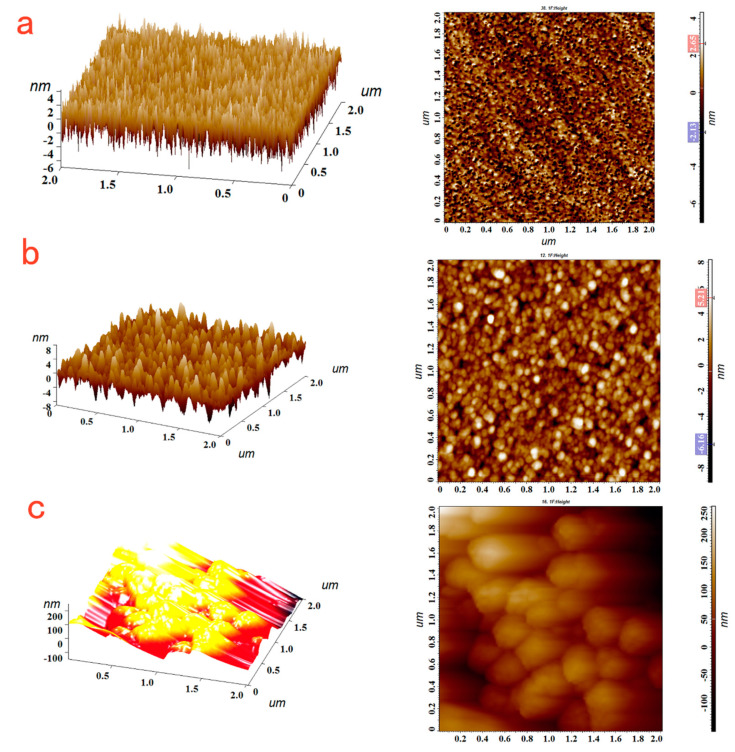
The AFM picture of the bare film (**a**); the AFM picture of the T2−OVA immobilized on the surface of the chip (**b**); the AFM picture of mAb−Fe_3_O_4_@SiO_2_@AuNPs binding to the surface of the chip (**c**).

To further confirm the immobilization of T2-OVA on the surface of the gold chip film, we carried out AFM image characterization. Figure 5a shows that the surface of the bare gold film was relatively smooth, and its root mean square roughness was 1.29 nm. After the binding of T2-OVA to the chip, the surface of the functionalized chip was ragged and its root mean square roughness increased into 1.81 nm, as shown in Figure 5b. It can be clearly observed that the mAb-Fe_3_O_4_@SiO_2_@AuNPs were well dispersed on the surface of the Au film bound with T2-OVA in Figure 5c. Additionally, its root mean square roughness was 132.32 nm.

### 3.3. T-2 Toxin Detection

**Figure 6:**
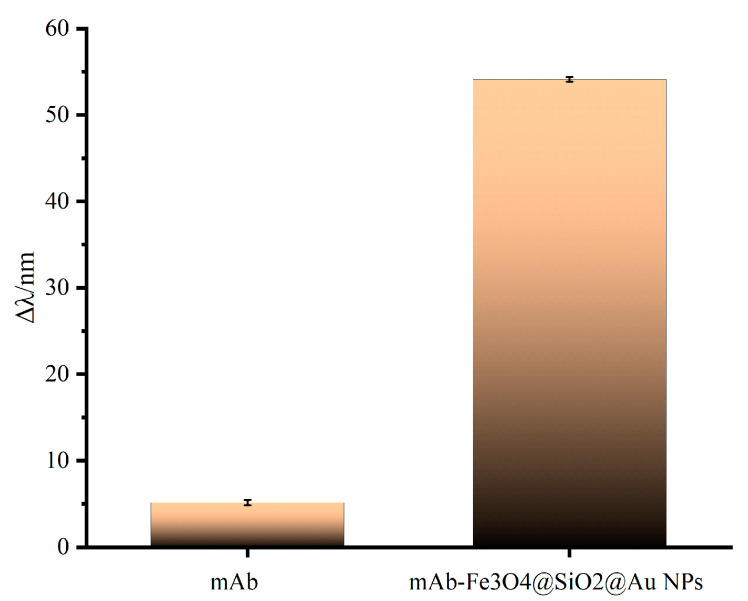
The SPR response of T-2 toxin monoclonal antibody and T-2 toxin monoclonal antibody modified with magnetic Fe_3_O_4_@SiO_2_@Au NPs.

In order to verify the amplification strategy of magnetic Fe_3_O_4_@SiO_2_@AuNPs, the SPR response was compared for the two detection strategies (the direct method and the direct competition method). T-2 toxin monoclonal antibody and antibody-coupled Fe_3_O_4_@SiO_2_@AuNPs conjugates [35] were adsorbed onto the surface of the sensing chip modified by T2-OVA under the same experimental conditions, respectively. As shown in Figure 6, when only T-2 toxin monoclonal antibody was injected, the resonance wavelength was red-shifted by 5.14 nm. However, when antibody-coupled Fe_3_O_4_@SiO_2_@AuNPs conjugates was injected, the resonance wavelength was red-shifted by 54.11 nm. When detecting the same concentration of T-2 toxin monoclonal antibody (15 μg/mL), the amplification strategy based on Fe_3_O_4_@SiO_2_@AuNPs amplified the SPR signal 10.53 times compared with the direct detection method. The causes for effective SPR amplification signals were mainly the higher refractive index and molecular mass of the Fe_3_O_4_@SiO_2_@AuNPs nanocomposites, the electronic coupling between the AuNP compound and the propagating surface plasmon wave close to the gold film [36], and the stems from the high sensitivity of the magnetic–plasmonic nanomaterials (Fe_3_O_4_@SiO_2_@AuNPs) to the refractive index in the solution [37]. In addition, the Fe_3_O_4_@SiO_2_@AuNPs compounds could separate and enrich the samples, efficiently reducing the background interference of complex samples, which was time-saving and convenient.

**Figure 7:**
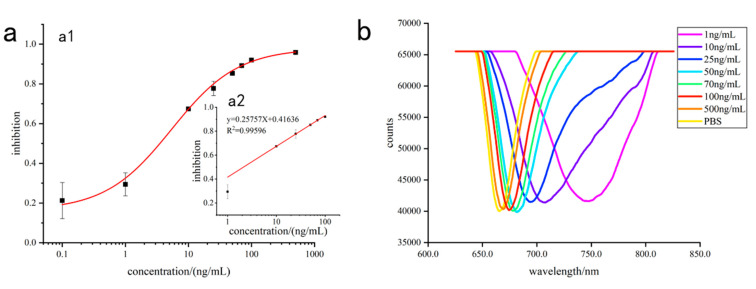
Direct competitive result of enhanced SPR response: direct competitive inhibition curve (**a1**) and calibration curve of T-2 toxin detection (**a2**); the SPR response curves of T-2 toxin at different concentrations (**b**).

To realize the sensitive detection of T-2 toxin, the optimal concentrations of anti-T-2 toxin monoclonal antibody and mAb-Fe_3_O_4_@SiO_2_@AuNPs were 15 μg/mL (Figure S3) and 2 mg/mL (Figure S4), respectively. A total of 2 mg/mL Fe_3_O_4_@SiO_2_@AuNPs was combined with 15 μg/mL of T-2 toxin monoclonal antibody; then, different concentrations of T-2 toxin were added to it; and finally, the mixture was injected into the sample chamber for detection on the surface of the functionalized chip. Before the mixture of T-2-mAb-Fe_3_O_4_@SiO_2_@AuNPs was introduced into the sample chamber, a certain amount of mAb-Fe_3_O_4_@SiO_2_@AuNPs first reacted fully with different concentrations of T-2 toxin. There were mAb-Fe_3_O_4_@SiO_2_@AuNPs without T-2 toxin in the solution. Then, a mixture of T-2-mAb-Fe_3_O_4_@SiO_2_@AuNPs was injected into the sample chamber on the surface of the functionalized chip. The remaining mAb-Fe_3_O_4_@SiO_2_@AuNPs were combined with T2-OVA on the gold film to produce an SPR response, so T2-OVA on the gold film competed with T-2 toxin to bind mAb-Fe_3_O_4_@SiO_2_@AuNPs. As shown in Figure 7b, the higher the concentration of T-2 toxin, the weaker the SPR response; in other words, the concentration of T-2 toxin was negatively correlated with the SPR response. When the substance to be tested was a negative sample (mAb-Fe_3_O_4_@SiO_2_@AuNPs), the SPR response value was R_0_, and the SPR response value of a certain concentration of T-2 toxin was R. The inhibition (%) was calculated using [(R_0_-R)/R_0_] × 100%. A direct competitive inhibition curve and inhibition regression curve are shown in Figure 7a. In the range of 1 ng/mL~100 ng/mL, the inhibition had a good linear relationship with the concentration of T-2 toxin. The regression equation was y = 0.25757x + 0.41636 (R^2^ = 0.99596). According to the principle of S/N ≥ 3, the limit of detection (LOD) was calculated to be about 0.57 ng/mL (LOD = 3 × standard deviation/slope). Compared with the methods for the detection of T-2 toxin in Table 1, the SPR sensor based on the Fe_3_O_4_@SiO_2_@Au nanoparticle magnification strategy had a lower limit of detection, which indicates the successful construction of a sensitive SPR sensor for T-2 toxin detection.

### 3.4. Specificity and Repeatability of the Strategy

**Figure 8:**
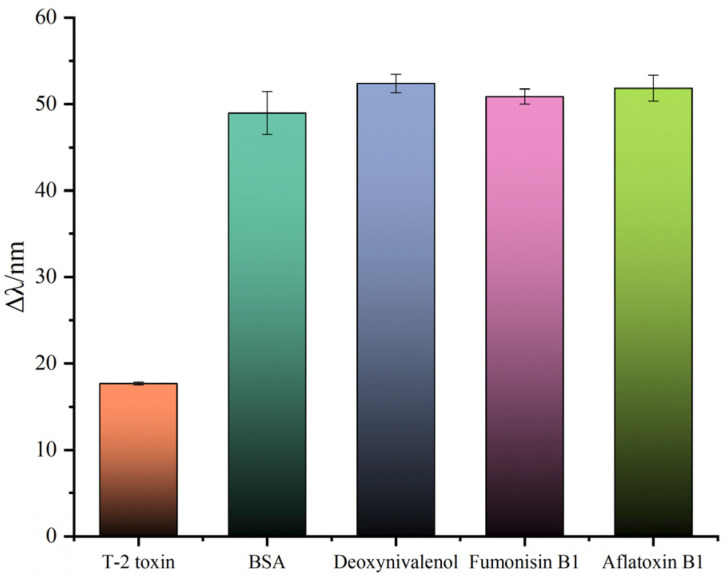
Specificity analysis of SPR biosensors.

To further evaluate the specificity of the SPR sensor using this method to detect T-2 toxin, deoxynivalenol, fumonisin B1, aflatoxin B1 and BSA were selected and measured under the same conditions as above for the detection of T-2 toxin. The concentrations of these analogues were 10 ng/mL when tested, and the experimental results are shown in Figure 8. Because of the use of the direct competition method to detect these analogues, only when T-2 toxin existed in the solution was the SPR resonance wavelength red shifted by about 17 nm, and the resonance wavelengths of other analogues were about 50 nm, indicating that the use of this method to detect T-2 toxin has strong specificity. At the same time, different concentrations of T-2 toxin (70 ng/mL, 50 ng/mL and 10 ng/mL) were repeatedly detected three times under the same experimental conditions. We can observe from Table 2 that the theoretical value was basically consistent with the test value, and the RSD value ranged from 0.90% to 3.97%. These results were sufficient to show that this method has good repeatability and selectivity.

### 3.5. Analysis of Actual Samples

In order to evaluate the feasibility and reliability of the SPR sensor with Fe_3_O_4_@SiO_2_@Au nanoparticles as the amplifying signal element for the detection of T-2 toxin in practical application, a spiked recovery test was carried out with milk, soil and rainwater as actual samples. Different concentrations of T-2 toxin were added to the pretreated milk, soil and rainwater samples to make concentrations of 25 ng/mL, 10 ng/mL and 3 ng/mL, and SPR sensing detection was performed using the direct competition method. The results in Table 3 show that the recovery range was 83.12~108.4%, and the RSD value was 0.61~15.38%. This detection method has good accuracy and can be used for the detection of T-2 toxin residues in actual samples.

## 4. Conclusions

We proposed a strategy to amplify SPR signals based on Fe_3_O_4_@SiO_2_@Au nanoparticles, and used the direct competition method for the real-time online detection of T-2 toxin. This method has the advantages of high sensitivity, strong specificity, and label-free and real-time monitoring. At the same time, the introduction of Fe_3_O_4_@SiO_2_@Au nanoparticles not only effectively enhances the response of SPR and improves the sensitivity of SPR sensing to detect small molecules, but can also quickly enrich and separate target components from actual samples under an external magnetic field; moreover, it can greatly shorten sample pretreatment time. Under optimal conditions, T-2 toxin had a good linear relationship in the range of 1 ng/mL~100 ng/mL, and the detection limit was 0.57 ng/mL. This work also shows the enormous potential of SPR sensors based on the Fe_3_O_4_@SiO_2_@Au nanoparticle amplification strategy for small molecule detection and disease diagnosis.

## Figures and Tables

**Table 1:** Comparison of the methods for the detection of T-2 toxin.

Analytical Methods	Linear Range	Limit of Detection	Reference
Fluorescent aptasensor	10~180 ng/mL	7.23 ng/mL	[29]
Electrochemical sensor	1.12 nM~2.12 μM	150 ng/mL	[38]
SPR screening assay	250~2000 ng/mL	6 ng/mL	[39]
Lateral-flow immunochromatographic assay (LFIA)	<400 ng/mL	230 ng/mL	[30]
SPR sensor based on Fe_3_O_4_@SiO_2_@Au nanoparticles	1~100 ng/mL	0.57 ng/mL	This work

**Table 2:** Repeatability analysis of SPR biosensor.

Theoretical Value (ng/mL)	Test (ng/mL)	RSD (%)
10	9.88	0.90
50	50.60	3.97
70	71.27	2.66

**Table 3:** Detection of T-2 toxin in real samples.

Sample	Spiked (ng/mL)	Test (ng/mL)	Recoveries (%)	RSD (%)
Milk	25	24.02	96.07	13.86
	10	9.83	98.26	3.61
	3	2.50	83.12	2.88
Soil	25	24.89	99.55	15.38
	10	9.74	97.45	7.87
	3	2.77	92.47	2.11
Rain water	25	22.91	91.65	5.58
	10	10.84	108.41	0.67
	3	2.79	92.93	0.61

## Data Availability

Not applicable.

## Supplementary Materials

The supplementary data associated with this article can be found in the online version at https://www.mdpi.com/article/10.3390/s23063078/s1.
