# Supplementary material for: Surface Plasmon Resonance Sensor Based on Core-Shell Fe3O4@SiO2@Au Nanoparticles Amplification Effect for Detection of T-2 Toxin
Source: Sensors (Basel). 2023 Mar 13;23(6):3078. doi: 10.3390/s23063078 (PMC10055945; doi:10.3390/s23063078)
Supplement: Supplementary file 1 [file sensors-23-03078-s001.zip › sensors-2221809-supplementary.pdf]

Supplementary Materials

Surface Plasmon Resonance Sensor Based on Core-Shell $Fe_3O_4@SiO_2@Au$ Nanoparticles Amplification Effect for Detection T-2 Toxin

2.1 **Reagents and Instruments**

Monoclonal antibody against T-2 toxin and T2-OVA were purchased from Shandong Landu Biotechnology Co., Ltd. T-2 toxin, Deoxynivalenol, Fumonisin B1and Aflatoxin B1 were purchased from Pribolab Pte. Ltd. (Singapore). poly (4-styrenesulfonic acid-co-maleic acid) sodium salt (PSSMA), Sodium acetate, ethylene glycol, $FeCl_3 \cdot 6H_2O$, diiodomethane (n=1.74) , 3-mercatopropionic acid, ethanol, TEOS,$NH_3 \cdot H_2O$,3-Aminopropyltriethoxysilane (APTES), Hydrogen tetrachloroaurate (HAuCl4),N-hydroxysuccinimide (NHS) and 1-ethyl-3-(3-dimethylaminoropyl)carbo-diimide hydrochloride(EDC) were purchased from Macklin Biochemical Co., Ltd(china), Bovine serum albumin(BSA) was purchased from Shanghai Acmec Biochemical Co., Ltd.

The zeta potential data of Fe_3O_4 particles, $Fe_3O_4@SiO_2$ particles, $Fe_3O_4@SiO_2$-NH2 particles and $Fe_3O_4@SiO_2@Au$ particles were accessed on the Zetasizer Nano-ZS nano analyzer (Malvern, UK). Determination of UV-Vis Absorption Spectra by Ultramicro Nucleic Acid Protein Quantitative Instrument (Thermo Fisher, USA). The AFM images were obtained by an automatic probe scanning microscope (NT-MDT, Russia).

2.2 **Construction of the SPR sensor**

The SPR sensor used in this paper was self-assembled by the experimental group[1]. The SPR analyzer is based on the Kretschmann structure SPR sensing model[2] to excite the surface plasmon effect by attenuated total reflection (ATR)[3]. The SPR sensing chip put on the optical prism, which is a triangular prism (45°/45°/90°, n=1.799 at 632.8 nm, Scott N-SF6). And the sample chamber was fixed to its chip. In order to enhance the coupling between prism and the sensing chip, the CH_2I_2 (n=1.74) was dropped on the prism and under the chip. Eventually, the sample chamber, sensing Au film and prism are fixed together to form a sandwich structure in the Fig S1. The SPR sensor obtains continuous wavelengths from a tungsten halogen lamp (LS-1, Ocean Optics, USA), and these wavelengths through a quartz fiber, collimator, and linear polarizer to form a polarization parallel light. And then the *p*-polarized parallel light is incident to the prism at a fixed incident angle ($\theta = 13°$). The relationship between the wavelength and the reflectivity of the reflected light is detected at the interface of the prism-coupled metal film. The wavelength corresponding to the lowest reflectivity of the reflected light is the resonance wavelength(λ). Ultimately, the

reflected beam is transmitted to the spectrometer (USB 2000+, Ocean Optics, USA) through a collimator and quartz fiber, and the spectral data is recorded.

When SPR resonance occurs, the position of the resonance peak is related to the refractive index of the medium on the surface of the metal layer which changes with the mass of the attached biomolecules. During SPR sensing analysis, the ligand is first coupled to the surface of the gold chip and secondly the sample containing the analyte is injected into the sample cell, and then the ligand is combined with the analyte leading to the refractive index of the surface of the sensing chip changes, and the resonance peak changes accordingly.

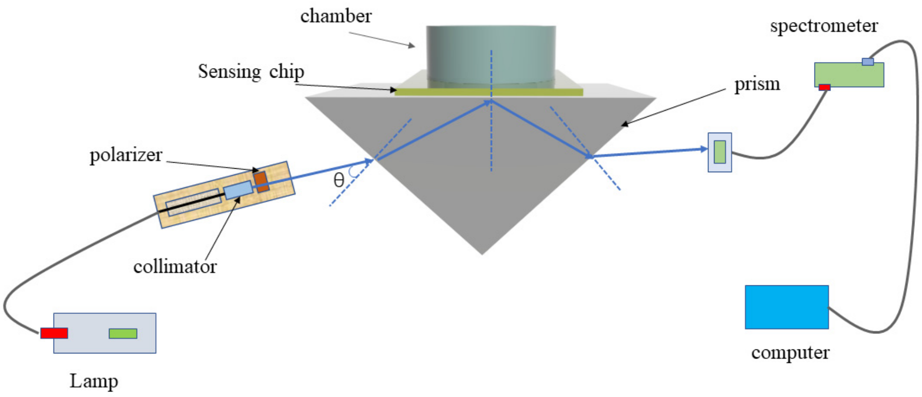

Figure S1. Schematic diagram of SPR sensing device

Immobilization of T2-OVA

The relationship between T2-OVA of different concentrations (50,100,150,200,250μg/mL) and SPR angle shift was shown in the Fig S2. The SPR signal was gradually increased along with concentrations of T2-OVA increasingly added. It is obvious that immobilization nearly attained saturation when concentration of T2-OVA was 200μg/mL. Therefore, the optimal fixed concentration of T2-OVA was 200μg/mL.

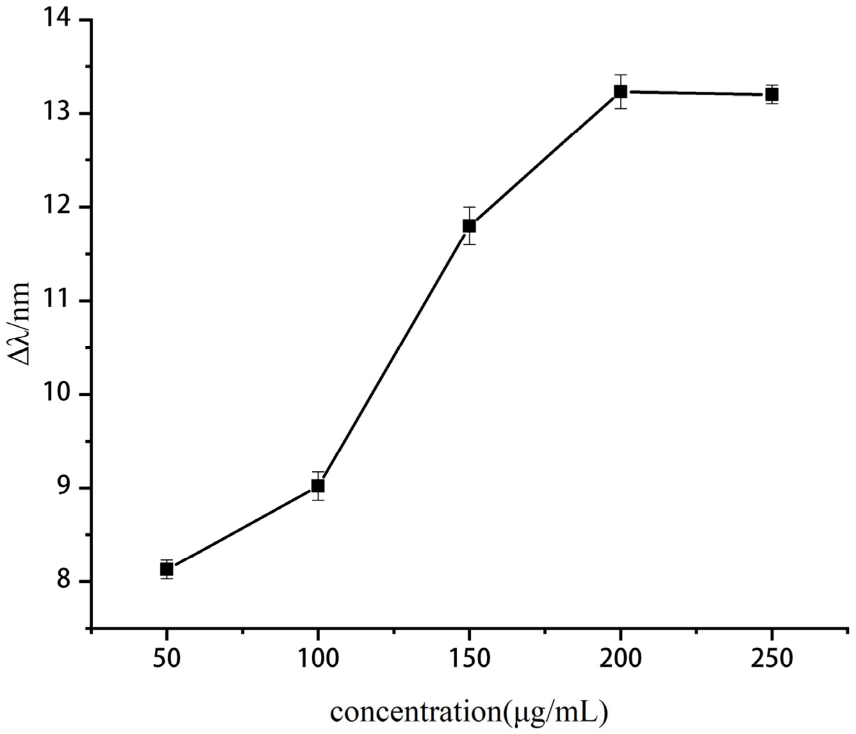

Figure S2. the SPR angle response of different concentrations of T2-OVA.

Selection of optimal T2-mAb concentrations

Different concentrations of T-2 toxin monoclonal antibodies (3μg/mL, 6μg/mL, 10μg/mL, 15μg/mL, and 18μg/mL) were mixed with 1mg/mL Fe_3O_4@SiO_2@Au NPs for 10h. and the conjugates（mAb- Fe_3O_4@SiO_2@Au NPs） were magnetic separation with an external magnetic field. The supernatant was taken to detect ultraviolet at 280 nm and the precipitate was redispersed with PBS to form a solution with the concentration of 1 mg/mL for SPR sensing detection. As shown in Table S1, the concentration of T2-mAb in the supernatant of T2-mAb was almost not until the concentration of T2-mAb was 15μg/mL . At the same time, when the concentration of T-2 monoclonal antibody was 15μg/mL, the change of SPR angle was the most obvious in the Fig S3. When the concentration of T2-mAb increases, it may lead to the increase of steric hindrance and the decrease of SPR angle change value. Furthermore, the optimal concentration of T2-mAb was 15μg/mL.

Table S1 Selection of optimal T2-mAb concentrations

Concentrations of T2-mAb (μg/mL)	Ultraviolet absorption of supernatant (280nm)
3	0
6	0.008
10	0
15	0.04
18	0.056

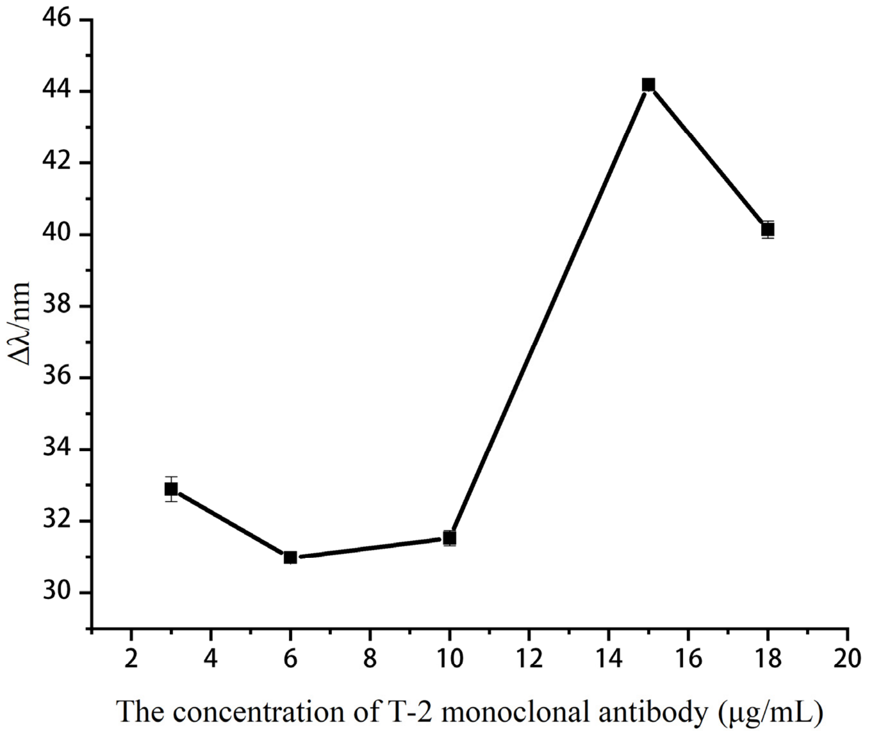

Figure S3. SPR angle shift of T2-mAb with different concentrations in Supernatant.

Selection of optimal Fe_3O_4@SiO_2@Au nanoparticles concentrations

Different concentration of Fe_3O_4@SiO_2@Au nanoparticles (0.5,1,2,3,4 mg/mL) were mixed with 15μg/mL T2-mAb in 4℃ for 10h. After one hour of blocking by 1% BSA, the mixtures were injected into the sample chamber on the surface of functionalized chip for sensing detection. It can be clearly observed in the Fig.S4 that as the concentration of mAb- Fe_3O_4@SiO_2@Au nanoparticles increased, the SPR angle change value gradually increased until its concentration reached 2mg/mL, and the SPR angle change was almost stable.

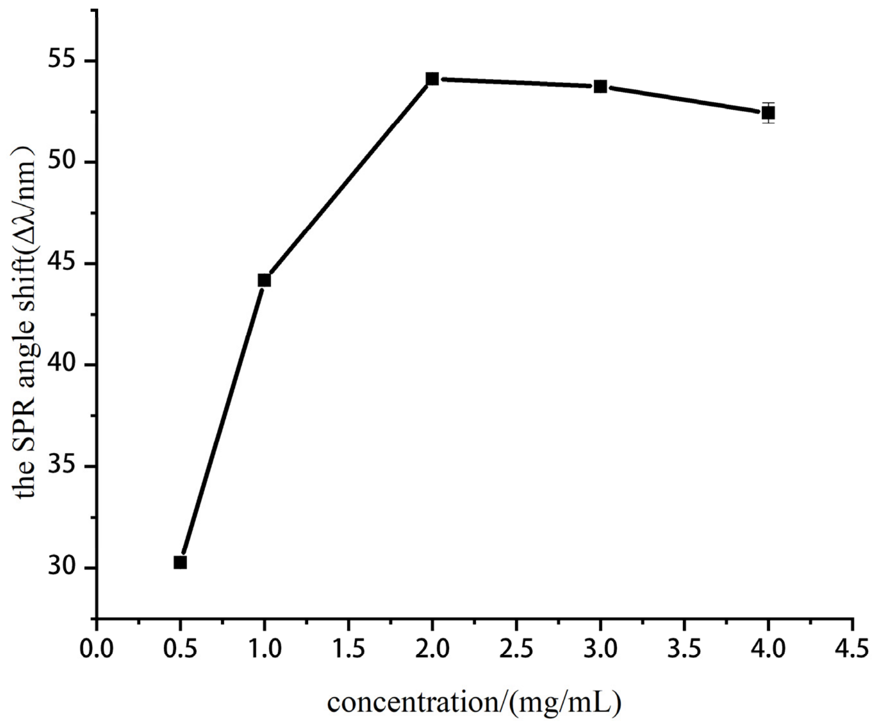

Figure S4. The SPR angle shift of mAb-Fe_3O_4@SiO_2@Au nanoparticles with different concentration.

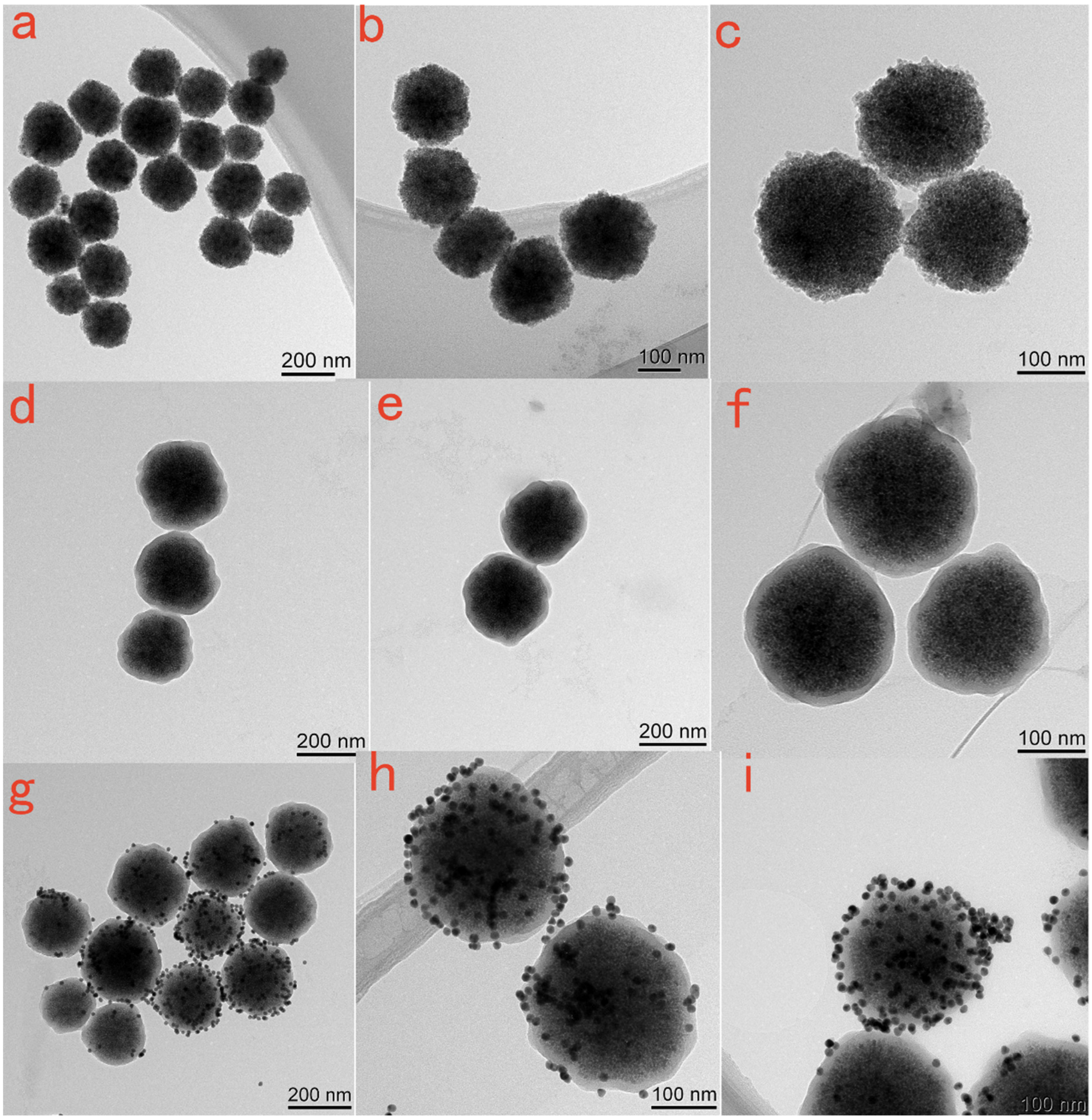

Figure S5. Characterization of nanoparticles: TEM images of (a, b, c) Fe_3O_4NPs, (d, e, f) Fe_3O_4@SiO_2NPs, (g, h, i) Fe_3O_4@SiO_2@AuNPs.

References

[1] X. Zhai, S. Cheng, H. Wang *et al.*, “Fast preparation of Fe3O4@polydopamine/Au for highly efficient degradation of tetracycline,” *Chemosphere,* vol. 285, pp. 131523, Dec, 2021.

[2] X. Jin, H. Li, S. Wang *et al.*, “Multifunctional superparamagnetic nanoshells: combining two-photon luminescence imaging, surface-enhanced Raman scattering and magnetic separation,” *Nanoscale,* vol. 6, no. 23, pp. 14360-70, Nov 6, 2014.

[3] S. Luo, Y. Liu, H. Rao *et al.*, “Fluorescence and magnetic nanocomposite Fe3O4@SiO2@Au MNPs as peroxidase mimetics for glucose detection,” *Anal Biochem,* vol. 538, pp. 26-33, Dec 1, 2017.
